# Palliative Thoracic Radiotherapy in the Era of Modern Cancer Care for NSCLC

**DOI:** 10.3390/cancers16173018

**Published:** 2024-08-29

**Authors:** Lucyna Kępka

**Affiliations:** Military Institute of Medicine—National Research Institute, 04-141 Warsaw, Poland; lucynak618@gmail.com

**Keywords:** palliative thoracic radiotherapy, non-small cell lung cancer, immunotherapy, targeted therapy, chemotherapy, dose and fractionation schedules

## Abstract

**Simple Summary:**

Radiotherapy is recognized as an effective tool for palliating symptoms associated with loco-regional growth of NSCLC. Most evidence regarding the use of palliative thoracic radiotherapy predates the new treatment era guided by molecular tumor characteristics and the utilization of modern radiotherapy technologies. The improved prognosis of disseminated NSCLC often complicates the distinction between palliative and curative radiotherapy. This review discusses dose and fractionation schedules, timing of radiation, treatment volumes, combinations with other therapies, and the toxicity of palliative thoracic radiotherapy within this evolving therapeutic landscape.

**Abstract:**

Palliative thoracic radiotherapy provides rapid and effective symptom relief in approximately two-thirds of NSCLC patients treated. In patients with poor performance status, the degree of palliation appears unrelated to the radiation dose or fractionation schedule. Conversely, in patients with good performance status, higher radiation doses administered over longer periods have shown modest survival benefits. These findings stem from studies conducted before the advent of immunotherapy and targeted therapy in clinical practice. Currently, there are no large prospective studies specifically dedicated to palliative radiotherapy conducted in this new treatment era. Modern radiotherapy technologies are now widely available and are increasingly used for palliative purposes in selected patients, reflecting the expanded array of therapeutic options for disseminated NSCLC and improved prognosis. Some traditional tenets of palliative thoracic radiotherapy, such as the improvement of overall survival with a protracted radiation schedule and the use of simple, cost-effective radiation techniques for palliative purposes, may no longer hold true for patients receiving immunotherapy or targeted therapy. The application of IMRT or SBRT in the context of palliative radiotherapy for NSCLC is not yet sufficiently explored, and this is addressed in this review. Moreover, new risks associated with combining palliative radiotherapy with these systemic treatments are being explored and are discussed within the context of palliative care. The optimal timing, doses, fractionation schedules, and treatment volumes for radiotherapy combined with immunotherapy or targeted therapy are currently subjects of investigation. In emergencies, radiotherapy should be used as a life-saving measure without delay. However, for other indications of palliative thoracic radiotherapy, decisions regarding doses, timing relative to systemic treatments, and treatment volumes should be made in a multidisciplinary context, considering the patient’s prognosis, anticipated outcomes, and access to potentially effective treatments. We still lack robust data from prospective studies on this matter. This review examines and discusses available evidence on the use of palliative thoracic radiotherapy within the framework of modern treatment strategies for NSCLC.

## 1. Introduction

Radiotherapy plays a well-established role in the management of non-small-cell lung cancer (NSCLC) across all stages of the disease. It is recommended as a curative treatment for patients with early-stage NSCLC who are medically inoperable or decline surgery, and it is also used as part of combined therapy or occasionally as a sole modality for locally advanced NSCLC. Recently, there has been a rapid evolution of the use of curative radiotherapy for oligometastatic NSCLC. Evidence suggests that local treatment improves survival in oligometastatic NSCLC, as demonstrated in the SINDAS trial, which included patients with EGFR-mutated NSCLC [1]; the SABR-COMET trial [2], which included 18 patients with NSCLC; and two small clinical trials on consolidative local treatment in NSCLC [3,4]. However, patients in these trials did not receive immunotherapy. Thus, the role of radiotherapy in the context of the routine use of these new agents in metastatic NSCLC remains under investigation.

Symptoms related to local tumor growth, such as cough, dyspnea, hemoptysis, dysphagia, superior vena cava syndrome (SVCS), or pain, affect approximately 75–85% of patients with locally advanced or metastatic NSCLC referred for radiotherapy [5,6]. Effective management of these cancer-related symptoms is crucial, and radiotherapy is widely recognized as an effective treatment option. Prospective studies have shown that palliative thoracic radiotherapy alleviates symptoms in 65–77% of patients [7].

The evidence demonstrating the high effectiveness of palliative thoracic radiotherapy largely predates the era of new treatment strategies for NSCLC, such as targeted therapy guided by molecular tumor characteristics and immunotherapy, either as monotherapy or in combination with chemotherapy. Additionally, many modern radiotherapy techniques, such as three-dimensional conformal radiotherapy (3D-CRT), intensity-modulated radiotherapy (IMRT), and stereotactic body radiotherapy (SBRT), were not available or widely used for palliative purposes in the trials that established the role of palliative thoracic radiotherapy for NSCLC. While the efficacy of palliative radiotherapy as a longstanding treatment method is unquestioned, the introduction of new treatment technologies raises questions regarding its optimal use in the current therapeutic landscape. These include considerations about the timing of radiotherapy in relation to new systemic treatments, the safety of combining radiotherapy with these new agents, and optimal schedules and doses of palliative thoracic radiotherapy. Many issues related to the combination of palliative radiotherapy with immunotherapy or targeted therapy remain unresolved. These uncertainties regarding the use of radiotherapy with immunotherapy were highlighted in two recently published surveys [8,9]. In the German survey, 51 radiation oncologists assessed their knowledge about immunotherapy in cancer management through an online questionnaire. They were asked to rate their knowledge on the use of immunotherapy on a scale from 1 to 10, where 1 referred to “very limited knowledge” and 10 to “excellent knowledge”. Of the respondents, 49% scored their knowledge below 6 [8]. Similarly, in a Dutch survey (27 radiation oncologists, 10 medical oncologists, and 17 pneumonologists), more than half of the participants reported insufficient knowledge regarding the combination of targeted therapy or immunotherapy with radiotherapy [9]. This reflects the need for evidence-based guidelines on the clinical use of these new combinations, particularly in the more commonly used palliative setting.

Furthermore, advancements in systemic treatments have led to prolonged survival and broader access to therapeutic agents, complicating the distinction between patients suitable for curative versus palliative radiotherapy. Although cancer registries do not allow for a precise estimation of overall survival (OS) after palliative radiotherapy, and there are no large prospective trials evaluating the outcomes of palliative radiotherapy in the era of immunotherapy and targeted therapy, it is assumed that an improvement in prognosis for such patients exists. Data from the Surveillance, Epidemiology, and End Results (SEER) registry indicate that 2-year cancer-specific survival improved from 26% among men diagnosed with NSCLC in 2001 to 35% among those diagnosed in 2014. A similar improvement over the same period was observed in women [10]. In a French monocentric cohort of 2193 patients with metastatic lung cancer (NSCLC and SCLC) treated between 2000 and 2020, 2-year overall survival improved for NSCLC according to the treatment period: 26.4%, 39.6%, and 43.4% for 2000–2009, 2010–2017, and 2018–2020, respectively (*p* = 0.0004). Such an improvement was not demonstrated for SCLC over time. This suggests a real impact of the approval of new treatment strategies (2010: Targeted therapy; 2018: Immunotherapy) on the prognosis of metastatic NSCLC [11]. Thus, improved survival with the advent of these new treatment strategies in some patients referred for palliative radiotherapy should be acknowledged. With longer survival, these patients are at greater risk of local failure and complications from palliative radiotherapy. If patients are receiving treatment not only for symptom relief but also for durable local control or prevention of symptoms, the fractionation schedule, total dose, and radiotherapy technique should be individualized. Hypofractionation (i.e., the use of doses per fraction higher than 2.2 Gy, usually between 3–5 Gy) is preferred in palliative radiotherapy compared with conventional fractionation (daily doses of 2 Gy). Large doses per fraction are convenient for patients and result in quick palliation; however, they lead to more severe late effects, which may have serious consequences for survivors. To reduce the risk of damage to late-responding normal tissues, a decrease in total dose is needed, which could potentially reduce tumor control probability. However, the shorter overall treatment time may compensate for this negative effect, and hypofractionation may be used as a convenient way of accelerating treatment [12]. With appropriate case selection, the increased cost of protracted fractionation or more conformal treatments can be justified where clinical benefit is expected. Advanced radiation techniques such as 3D-CRT, compared to 2-dimensional approaches, improve treatment conformality, thereby potentially reducing toxicity. However, while historically less suited for palliative radiotherapy, modern technologies such as IMRT and SBRT are increasingly viable options in radiation departments with expanded resources. The potential radiobiological benefit of high fractional doses delivered by SBRT, especially in combination with immunotherapy, will be discussed.

This review aims to present available evidence and highlight unresolved questions regarding the use of palliative thoracic radiotherapy in the context of modern treatment strategies for NSCLC.

## 2. Palliative Thoracic Radiotherapy Schedules: For Symptomatic and Asymptomatic Patients

The prerequisite for the use of palliative radiotherapy is, by definition, the presence of symptoms that can be relieved by radiation. In the case of palliative thoracic radiotherapy for NSCLC, these symptoms typically result from intrathoracic growth of primary tumors or regional (nodal) metastases, commonly presenting as dyspnea, cough, hemoptysis, superior vena cava syndrome (SVCS), or pain. Occasionally, palliative radiotherapy schedules are also considered in asymptomatic patients as a preventive measure, especially when the tumor’s location poses a risk of developing bothersome symptoms. Preventing symptoms is not typically viewed as a primary goal of palliative care. However, the WHO’s definition of palliative care extends beyond symptom alleviation in incurable diseases to include strategies aimed at preventing symptom occurrence or worsening [13]. Table 1 outlines various clinical scenarios where palliative thoracic radiotherapy may be integrated into cancer management.

The use of palliative radiotherapy in asymptomatic patients has been a topic of debate. Evidence does not support the notion that thoracic palliative radiotherapy prevents the onset of symptoms. In fact, two randomized trials found no benefit from immediate palliative radiotherapy in terms of symptom control among asymptomatic or minimally symptomatic patients who were not receiving systemic treatment. One study randomized 230 such patients to receive immediate radiotherapy with a single fraction of 10 Gy or two fractions of 8.5 Gy compared to delaying radiotherapy until symptom progression. No significant differences were found in symptom control, overall survival, or quality of life (QoL). Importantly, 56% of patients who did not receive immediate radiotherapy did not require thoracic radiotherapy until death [14]. A post hoc analysis of a Norwegian trial, in which patients were randomized to three different palliative thoracic radiotherapy schedules and were stratified according to the presence of symptoms, demonstrated no improvement in long-term symptom control in patients without thoracic symptoms at baseline compared with symptomatic patients, with worsened QoL observed in the weeks following radiotherapy [15]. Therefore, the idea that palliative radiotherapy prevents symptom occurrence lacks evidence. However, these trials were conducted before the era of immunotherapy or targeted therapy, leaving uncertainty about how radiotherapy in asymptomatic patients receiving these treatments affects symptom control.

Considering the challenged role of radiotherapy in symptom prevention, questions arise regarding its impact on survival. Can palliative radiotherapy schedules prolong survival in patients who are not candidates for systemic treatment or curative radiotherapy due to frailty or extensive thoracic disease? Systematic reviews of randomized trials suggest that such benefits exist for selected patients. A review of 13 randomized trials demonstrated that higher radiation doses led to improved survival rates in patients with good performance status (PS). Doses equal to or higher than a biologically equivalent dose (BED) of 35 Gy showed approximately a 5% improvement in 1-year survival compared to lower doses (primarily single doses), *p* = 0.002 [7]. Similar conclusions were drawn in a Cochrane systematic review of 14 studies, where higher dose regimens correlated with improved survival in subpopulations of patients with good PS [16]. However, this survival benefit with higher doses was accompanied by significantly higher esophageal toxicity [7,16]. Therefore, immediate palliative radiotherapy of approximately 30 Gy in 10 fractions should be considered for asymptomatic patients with good PS who are unable to receive systemic therapy or curative radiation doses.

Experts’ guidelines recommend the use of radiotherapy for oligoprogression or oligorecurrence during targeted therapy or immunotherapy in metastatic patients, even in the absence of symptoms, although robust evidence supporting prolonged survival with the addition of radiotherapy in patients with oligoprogression on immune checkpoint inhibitors (ICI) is lacking. Ablative radiotherapy is specifically recommended upon first progression under first-line epidermal growth factor receptor (EGFR) tyrosine kinase inhibitors (TKI), anaplastic lymphoma kinase (ALK), ROS1 inhibitors, or ICI, with continuation of the first-line agent [17,18]. The rationale behind this recommendation is that lesions progressing despite inhibitor therapy may exhibit different biological behaviors due to tumor heterogeneity or acquired resistance to inhibitors, while the systemic effect of the drug on sensitive clones may still be active. SBRT is preferred in these cases for its ability to deliver high curative doses. However, in scenarios where SBRT is not feasible due to lesion location or size, lower palliative doses may also be considered, although there is insufficient evidence to fully support this approach. In the randomized SINDAS study, upfront radiotherapy with moderate doses (25–40 Gy in fractions) administered to all sites of oligometastatic disease in EGFR-positive NSCLC before TKI therapy was shown to improve median OS compared to TKI therapy alone (TKI only: 17.4 months; TKI + radiotherapy: 25.5 months; *p* < 0.001) [1]. This prompts reconsideration of using lower doses of radiotherapy in combination with targeted therapy, and potentially immunotherapy, in asymptomatic patients when ablative doses are not feasible. A Canadian prospective observational single-arm study (NCT03705806) is currently investigating stage IV NSCLC patients routinely treated with ICI approved by Health Canada. All selected patients undergo thoracic radiotherapy with a palliative schedule of 30 Gy in 10 fractions, regardless of symptom presence. The primary endpoint of this study is toxicity, but it also aims to shed light on symptom prevention in these patients. No other palliative thoracic radiotherapy schedules in combination with immunotherapy for symptomatic or asymptomatic patients were identified in our search.

Should thoracic radiotherapy of the primary tumor +/− regional lymph node metastases in oligometastatic disease in asymptomatic patients be considered as a palliative or curative approach? This is debatable. Based on an ESTRO-ASTRO consensus, oligometastatic disease is currently defined as 1–5 metastatic lesions, with a controlled primary tumor being optional, but all metastatic sites must be safely treatable, with the expectation that the disease is amenable to a curative approach [19]. Thus, rather curative doses should be given in thoracic radiotherapy. Taking into account the generally poorer prognosis than in non-metastatic patients and the need for prolonged systemic treatment, hypofractionated schedules or SBRT, if technically feasible, are the preferred schemas.

## 3. Dose, Fractionation, and Techniques

A higher biological dose may prolong survival in patients with good PS, as previously mentioned. However, when the goal is symptom alleviation in cancer patients, reducing treatment time to a single fraction is an appealing option. During the 1990s and early 21st century, numerous prospective trials investigated the palliative effects of different doses and fractionation schedules. Despite heterogeneity across studies in terms of dose/fractionation schedules, patient characteristics, and outcome measures, these trials consistently showed that higher doses with more protracted radiotherapy schedules are equally effective for palliation compared to single or two-fraction treatments [6,20,21,22,23,24,25,26,27]. Systematic reviews of randomized trials on dose/fractionation in palliative thoracic radiotherapy for NSCLC confirm no significant differences in the palliative effects of different radiation doses. However, these reviews indicate that higher doses improve overall survival (OS) in patients with good PS, albeit at the expense of increased esophageal toxicity [7,16]. Overall, the toxicity associated with palliative radiotherapy is relatively low. A systematic review and meta-analysis comparing toxicity between palliative and radical radiotherapy acknowledged high heterogeneity in fractionation schedules and toxicity scoring methods. No treatment-related deaths were reported in palliative radiotherapy trials. Comparison of any grade of esophagitis between curative and palliative radiotherapy was possible in only two trials and showed a higher, albeit statistically insignificant, rate in curative versus palliative radiotherapy (35.4% vs. 26.6%, *p* = 0.06). Data for comparison of grade 3 or higher esophagitis between palliative and curative radiotherapy were lacking [28].

Can new technologies reduce the side effects of palliative radiotherapy? Traditional techniques like parallel-opposed (anterior-posterior) fields have historically been used in palliative radiotherapy due to lower treatment costs and patient comfort associated with reduced treatment machine time and preparation. However, newer techniques such as volumetric modulated arc therapy (VMAT) may offer shorter treatment times and greater convenience for patients. A small multicenter prospective study involving 35 NSCLC patients receiving palliative radiotherapy (39 Gy in 15 fractions, 20 Gy in 5 fractions, and 17 Gy in 2 fractions) for stage III and IV disease evaluated how 3D-CRT, minimizing esophageal doses, reduced esophagitis compared to historical cohorts. Fourteen percent of patients experienced grade 2 esophagitis during treatment, with resolution at the one-month follow-up [29]. A planning study on 15 lung cancer patients receiving palliative radiotherapy (30 Gy in 10 fractions) suggested that, compared to standard parallel-opposed beams, median esophageal doses were reduced from 16 to 7 Gy and the theoretical probability of symptomatic esophagitis was decreased from 13% to 2% [30]. A phase III trial provided evidence that IMRT reduces esophageal toxicity related to palliative radiotherapy for lung cancer. Ninety patients receiving 30 Gy in 10 fractions (p. 54) or 20 Gy in 5 fractions (p. 36) were randomized to standard radiotherapy with parallel-opposed pairs in the control arm or IMRT with intentional avoidance of the esophagus in the experimental arm. The incidence of symptomatic esophagitis was significantly lower with IMRT compared to standard treatment (2% vs. 24%, *p* = 0.002), particularly in patients receiving higher doses (30 Gy) [31].

SBRT for lung cancer delivers high ablative doses in a short treatment time, making it suitable for elderly or frail patients. Evidence of a survival benefit from the use of SBRT over conventional radiotherapy was demonstrated for early-stage lung cancer with a peripheral location [32]. High local control after SBRT for NSCLC results mainly from the fact that this technique provides dose distribution that makes it possible to prescribe a BED of 100 Gy or more. However, the applicability of SBRT in palliative thoracic radiotherapy for lung cancer is limited by the central location and large volume of tumors, for which SBRT can cause toxic effects, including life-threatening damage to central structures. Recently, the results of a nonrandomized phase 2 multicenter trial of individualized SABR (iSABR) enrolling 217 patients in the US and Japan demonstrated that minimizing the total dose, such as 60 Gy in 8 fractions for large tumors and central locations, provides excellent local control and low toxicity [33]. In such cases, IMRT may also be appropriate for administering a few fractions of hypofractionated radiotherapy at a relatively high total dose while minimizing doses to organs at risk like the esophagus. Combining new radiation technologies like SBRT or IMRT with immunotherapy, besides symptom relief or prevention, may eradicate all visible disease in oligometastatic disease or deliver a low total dose (e.g., 8 Gy in one to three fractions) to one disease site in polymetastatic disease. We do not have randomized trials evaluating survival depending on dose/fractionation in combination with ICI. Preclinical data indicate that hypofractionated schedules, with SBRT whenever possible, may induce stronger antitumor immunity via lower lymphocyte depletion than conventionally fractionated radiotherapy. In polymetastatic disease treated with ICI, the effect of the total dose may have a lower impact on survival than fraction size and irradiation volume [34].

Endobronchial brachytherapy (EBB) is used palliatively to rapidly relieve symptoms related to endobronchial obstruction causing pneumonitis, dyspnea, cough, or treat hemoptysis from bronchial tumors. Technological advancements in high dose rate, 3D planning, and remote afterloading since the 1990s have facilitated its use. The rapid decrease in doses outside the airways is considered the main advantage of this technique, especially in patients with impaired lung capacity. However, modern external-beam radiotherapy (EBRT) techniques like IMRT or SBRT, which rapidly reduce the dose outside the target area, offer a non-invasive alternative for such indications. Additionally, in palliative indications, the lesions are often large and associated with adenopathies, for which EBB is unsuitable. Systematic reviews comparing EBB with EBRT have found no benefit to using EBB as upfront treatment, recommending its use primarily for symptomatic patients with recurrent endobronchial disease after EBRT, provided it is technically feasible. Notably, these studies predominantly utilized older (two-dimensional) EBRT techniques [35,36]. Thus, currently, with newer technologies limiting the dose to the lung, we have even more possibilities for using upfront EBRT for patients with central tumors. Table 2 summarizes the advantages and disadvantages of respective technologies for palliative thoracic radiotherapy.

## 4. Palliative Thoracic Radiotherapy in Combination with Chemotherapy

Prospective data on adding chemotherapy to palliative radiotherapy remains limited. Concurrent chemoradiotherapy with curative intent is typically reserved for inoperable stage III patients with PS 0-1. In a Polish study, 99 patients ineligible for curative treatment due to the large volume of the disease, low FEV1, and PS 0-2 were randomized to receive either palliative radiotherapy (30 Gy in 10 fractions) alone or the same radiotherapy schedule combined with a third course of chemotherapy (cisplatin and vinorelbine). Noteworthily, the presence of tumor-related chest symptoms was also an inclusion criterion for the study. Symptom control was satisfactory and similar in both arms. Progression-free survival (PFS) and OS were significantly longer in the chemo-radiotherapy arm compared to radiotherapy alone, with median values of 7.3 vs. 4.7 months (*p* = 0.046) and 12.9 vs. 9.0 months (*p* = 0.0342), respectively [37].

In a Norwegian trial with similar eligibility criteria (except that symptom presence was not an inclusion criterion), 191 patients were randomized to receive either chemotherapy alone (four courses of carboplatin and vinorelbine) or the same chemotherapy regimen combined with radiotherapy (42 Gy in 15 fractions starting after the 2nd course of chemotherapy). Seventy-nine percent of patients had PS 0-1. Median OS was 9.7 months in the chemotherapy alone group and 12.6 months in the radio-chemotherapy group (*p* = 0.01) [38]. Based on these results, the panel of ASTRO experts recommends considering concurrent chemotherapy with palliative radiotherapy for a subset of stage III patients who are not candidates for curative treatment [39]. However, the lack of clear criteria for disqualification from radical treatment in both trials weakens these recommendations. Post hoc analysis of the Norwegian trial indicated that patients with PS 0-1 and large tumors derived the greatest benefit from combined treatment, whereas PS 2 patients did not [40]. Moreover, in the Polish trial, the palliative effect of treatment was comparable between patients receiving palliative radiotherapy alone and those receiving radio-chemotherapy.

In conclusion, a combination of hypofractionated radiotherapy—using doses lower than those intended for curative treatment but higher than standard palliative doses—may be considered for selected patients who are not suitable for curative management. For other patients, radiotherapy alone may be sufficient as a treatment strategy. However, the specific patient population that would benefit most from palliative radio-chemotherapy remains poorly defined. Importantly, both trials were conducted before the advent of immunotherapy and targeted therapy, which may now be accessible to patients disqualified from radical radiotherapy. Today, a combination of palliative radiotherapy with immunotherapy or targeted therapy is more frequently prescribed than radio-chemotherapy for this population [41]. The current research focus on combining palliative radiotherapy with these newer treatment modalities is discussed below.

## 5. Palliative Thoracic Radiotherapy in Combination with Immunotherapy

Stage IV NSCLC without driver mutations and with any PD-L1 expression in patients with PS 0-2 is an indication for immunotherapy alone or in combination with chemotherapy. Thoracic radiotherapy may be necessary for palliating tumor-related symptoms or for managing oligometastatic disease or oligoprogression during immunotherapy. Although strong data supporting survival benefits from radiotherapy in these cases are lacking, early-phase trials investigating the addition of radiotherapy to immunotherapy have shown promising results compared to historical series [18,42,43]. The impact of curative-dose radiotherapy combined with immunotherapy on survival exceeds the scope of this review. We will focus on all aspects of administering palliative thoracic radiotherapy combined with ICI, including symptomatic effects, timing relative to systemic treatments, dose/fractionation schedules, radiotherapy techniques, treatment volumes, and toxicity.

Prospective data on controlling tumor-related chest symptoms with radiation combined with ICI are limited. In a retrospective series involving 269 patients treated with nivolumab or pembrolizumab, 102 received palliative hypofractionated radiotherapy to various disease sites, including thoracic sites, either concurrently with or within 3 months of starting immunotherapy. Radiotherapy effectively controlled symptoms, but did not improve OS or PFS [44]. A systematic review of 13 prospective studies combining ICI with palliative radiotherapy (not restricted to thoracic sites) did not evaluate the symptomatic effects of radiotherapy due to incomplete and heterogeneous data [45]. As discussed earlier, all pre-immunotherapy randomized studies demonstrated that palliative outcomes, with symptom improvement rates of 65–70%, were independent of the radiotherapy schedule used (1–2 fractions vs. protracted schedules) [7]. Although the palliative effect of radiotherapy has not been studied in the context of immunotherapy, there is no rationale to question its efficacy for symptom relief, suggesting that the principle of palliative equivalence across different dose schedules remains valid for patients receiving ICI.

The choice of dose/fractionation schedule is crucial when combining radiotherapy with immunotherapy. Preclinical studies suggest that different dose/fractionation schedules have diverse immunologic effects on the tumor microenvironment and could impact radiotherapy outcomes when combined with immunotherapy. Conventional fractionation, with fractional doses around 2 Gy given over a period of 5–6 weeks, has a destructive effect on lymphocytes, especially when large volumes are treated [46,47,48]. Radiation-induced lymphopenia may negatively impact treatment outcome [49,50]. Conversely, hypofractionated radiotherapy (with doses ranging from 5 to 20 Gy per fraction) induces more T-cell activation within the tumor microenvironment, potentially enhancing efficacy compared to conventionally fractionated radiotherapy [51]. Moderate hypofractionation or single large doses are suitable for palliative purposes. Large ablative doses can be delivered using the SBRT technique, which allows for the delivery of high doses to small volumes with a rapid dose falloff outside the tumor, resulting in adequate sparing of normal tissues. This optimizes local control of treated sites and minimizes toxicity. SBRT is especially appealing when radiation is combined with immunotherapy due to its expected positive effects on the immune system, short overall treatment time, and limited tumor volume. When SBRT is administrated in oligometastatic disease in patients receiving ICI, it is expected that it can eradicate systemic therapy-resistant disease. However, many issues with combining SBRT and ICIs remain unresolved, such as optimal timing, lesion selection, and fractionation schedule (single vs. multiple large fractions), and are still under investigation. For purely palliative purposes, these concerns may be of lesser importance [52]. When SBRT is administered for oligometastatic disease in patients receiving ICIs, it is expected to potentially eradicate systemic therapy-resistant disease. However, in the palliative treatment of lung cancer, we often encounter large, central tumors that cause bothersome symptoms. While large ablative doses delivered via SBRT may be feasible, they could be too toxic for centrally located lung tumors [53,54,55]. Therefore, moderately hypofractionated schedules, such as 5 × 4 Gy, 10 × 3 Gy, or 15 × 2.8 Gy, or a single fraction of 8 Gy in patients with poor PS, may be more appropriate. For tumors without ultracentral locations (i.e., not in the main bronchi, trachea, esophagus, or major vessels) and with limited volume, SBRT with lower doses and a more protracted schedule may be considered.

The timing of palliative radiotherapy relative to ICI treatment is also important. In emergencies, such as massive hemoptysis, severe dyspnea due to airway obstruction, or superior vena cava syndrome (SVCS), radiotherapy should commence immediately as a life-saving measure, regardless of ICI use. Palliative radiotherapy may improve PS in patients with thoracic symptoms, enabling subsequent ICI prescription, which is only indicated for PS 0-2 patients [18]. However, the safety of combining radiation with ICI requires careful consideration. For patients with only minor or successfully managed thoracic symptoms, how to combine palliative radiation with ICI needs further investigation. A pooled analysis of 68 prospective trials from the US FDA database comparing patients who received radiation within 90 days before starting ICI therapy (1733 patients) to those who did not (13,956 patients) showed similar overall adverse effect rates, with no increased risk of high-grade adverse effects in the radiation group compared to those who received immunotherapy alone [56]. Concurrent administration of ICI with radiation raises additional concerns. A randomized trial evaluating the concurrent administration of pembrolizumab with radiotherapy (SBRT: 50 Gy in 4 fractions or hypofractionated radiotherapy: 45 Gy in 15 fractions) demonstrated the safety of this combined approach [57]. One small, randomized phase I trial included 37 treatment-naive patients with metastatic NSCLC receiving combined nivolumab and ipilimumab, comparing concurrent (SBRT + immunotherapy) versus sequential (SBRT followed by immunotherapy) radiotherapy. The safety of these two approaches was similar, with the concurrent approach not being more toxic than sequential treatment and being well tolerated by the entire cohort [58]. Another randomized trial, SABRseq, aimed to compare concurrent versus sequential administration of SABR with pembrolizumab in NSCLC patients, but was prematurely closed after including 13 patients due to poor accrual. No concerning safety signals were observed for either approach [59]. Concurrent administration of nivolumab with SBRT or conventional radiotherapy for metastatic NSCLC did not raise significant safety alerts in some small series [60,61,62,63]. In the recently published single-arm DOLPHIN trial, patients received radiotherapy (60 Gy) combined with concurrent and maintenance durvalumab immunotherapy. Pneumonitis or radiation pneumonitis of any grade occurred in 23 out of 34 evaluated patients (68%), with grades 3 or 4 in 4 patients (12%) [64]. Given the still-scarce and heterogeneous data on the safety of concurrent administration of radiation and ICI, it is prudent to avoid concurrent administration of these modalities for asymptomatic patients. The European Organisation for Research and Treatment of Cancer (EORTC)-ESTRO OligoCare consortium recommends avoiding same-day administration of SBRT and ICI. To mitigate potential risks, a minimum ICI interruption of one week before and after SBRT is advised [65].

The irradiation volume in combination with immunotherapy is actively investigated. For palliative radiotherapy, irradiating only symptomatic tumor extensions, such as in SVCS cases, may be reasonable. However, including all disease sites to achieve optimal local control is recommended for oligometastatic disease. ICIs target microscopic disease, while SBRT targets gross disease foci, potentially containing ICI-resistant clones, thereby prolonging the response to systemic treatment in oligometastatic disease [65,66,67]. In polymetastatic disease, determining which lesion to irradiate is complex. The decision depends on the response to ICIs and clinical judgment regarding the risk of symptomatic lesion progression. Irradiating a large volume may induce immunosuppression by depleting lymphocytes, potentially reducing the immunomodulatory effects of the drugs. Most studies on polymetastatic disease combine SBRT directed at one metastatic site with immunotherapy to explore the abscopal effect (response to immunotherapy outside the irradiated volume) [68]. However, defining which irradiated lesion will yield the strongest immunogenic activity, as seen in the abscopal effect, remains challenging. A pooled analysis of two randomized trials evaluating pembrolizumab with radiotherapy to one metastatic site showed a significantly higher abscopal response rate in the combined treatment group compared to pembrolizumab alone (41.7% vs. 19.7%, *p* = 0.0039) [69]. The choice of target lesion for palliative purposes is dictated by the symptoms presented. In cases of minor symptoms, decisions regarding whether to include a lesion, a portion of the lesion, or all gross disease foci should be made individually, considering the risk of side effects, disease volume, and treatment duration. If ICI treatment is paused during radiotherapy, the duration of radiotherapy is relevant. Radiotherapy should be completed within two weeks to ensure timely resumption of ICI.

The use of corticosteroids during immunotherapy is controversial. Clinical trials typically exclude patients receiving corticosteroids. However, palliative radiotherapy for chest symptoms often requires concurrent steroid administration to reduce tumor-related edema, inflammation, or obstruction. A study evaluating NSCLC patients receiving >10 mg vs. ≤10 mg of prednisone during immunotherapy found that those receiving >10 mg for cancer-related palliative purposes had significantly shorter progression-free survival (PFS) and overall survival (OS) compared to those receiving ≤10 mg or no steroids [70]. A systematic review and meta-analysis further supported these findings, showing that patients taking steroids during ICI treatment had an increased risk of disease progression and death compared to non-users [71]. However, the negative impact of steroids on PFS and OS was mainly observed in patients taking steroids for cancer-related symptom management, not for other reasons, such as the management of adverse events. Conversely, caution should be exercised with unnecessary corticosteroid prescriptions in patients receiving ICIs, as there are data on the negative impact of steroid use on ICI response. In a retrospective analysis of 475 patients with advanced solid tumors treated with ICI monotherapy from 2015 to 2022, patients exposed to steroids within 30 days before the first cycle of ICI had a lower objective response rate (20.3% vs. 36.7%, *p* < 0.01). A similar decrease in objective response rate was observed in patients taking steroids within the first 90 days of treatment (25.7% vs. 37.7%, *p* = 0.01). Interestingly, steroid exposure after 6 months of ICI was not associated with worse survival outcomes [72]. Therefore, steroids should be prescribed during palliative radiotherapy in patients receiving ICIs if clinically indicated to alleviate symptoms related to edema or tumor obstruction, but caution should be exercised regarding their prophylactic use.

## 6. Palliative Thoracic Radiotherapy in Combination with Targeted Therapy

Distinct biological characteristics and the high response rates associated with oncogene-addicted NSCLC complicate the distinction between palliative and curative radiotherapy. Consequently, high radiation doses, including ablative doses via SBRT, are often administered even for disseminated disease. Molecularly targeted therapies for stage IV oncogene-addicted NSCLC, although offering high response rates as initial treatment, do not cure the disease. Virtually all tumors will progress due to acquired drug resistance. For osimertinib, a third-generation, irreversible EGFR-TKI that selectively inhibits both EGFR-TKI-sensitizing and EGFR T790M resistance mutations, progression-free survival is approximately 18 months, compared with about 10 months for first- and second-generation TKIs [73,74,75]. This inevitable progression results from tumor heterogeneity in lung cancer, which is related to genomic instability, changing access to oxygen, cellular redistribution during therapy, and clonal evolution. Clonal evolution refers to sequential genetic alterations that promote successive generations of clones with higher proliferative potential, i.e., with acquired resistance to the drug, or is related to the baseline heterogeneity of the tumor, in which a minor population of cells with different genetic characteristics (e.g., resistance genes) proliferates over time, leading to tumor progression [76,77]. This means that some parts of tumors may remain sensitive to the used agent, but there is an urgent need for therapy to halt the progression of escaping therapy disease foci. This is where local therapy, commonly radiotherapy, plays a role in oligoprogressive disease, with the progressing lesion treated with radiation while continuing the currently employed inhibitor on sensitive tumor clones. Experts’ guidelines recommend continuing targeted therapy beyond progression when local treatment can be applied [17]. This approach also helps to avoid the flare phenomenon (rapid tumor progression due to regrowth of TKI-sensitive clones), which is observed in 20% of patients upon interruption of effective targeted therapy [78]. A similar rationale applies to the use of radiotherapy for oligopersistence, which refers to oncogene-addicted NSCLC diagnosed initially as oligometastatic. The proliferation of TKI-resistant clones in residual disease will lead to recurrence at the original disease sites and potentially to distant sites in about 60% of patients [79]. Radiotherapy directed at persistent disease sites aims to eradicate these resistant tumor cells and prevent or delay progression. Recently, upfront radiotherapy for oligometastatic disease in EGFR-positive NSCLC before TKI therapy has shown benefits for patients receiving radiotherapy compared to those treated with targeted therapy alone [1]. However, radiotherapy for oligoprogression, oligopersistence, or as upfront therapy often involves curative doses. Our focus is on the use of radiotherapy for palliative purposes.

EGFR TKIs, ALK inhibitors, and ROS1 inhibitors generally have milder toxicity profiles compared to chemotherapy. However, caution is warranted due to the radiosensitizing effect of TKIs, which can potentiate toxicity when combined with radiotherapy. Approximately 70% of patients receiving EGFR TKIs experience dermatologic side effects, with grade 3 or higher toxicity occurring in 2–16% of cases [80]. An increased rate of radiodermatitis has been demonstrated in patients treated with cetuximab when given concomitantly with radiotherapy compared to radiotherapy alone in a randomized phase 3 trial for locoregionally advanced head and neck cancer [81]. However, this indication involves large treatment volumes and high curative doses, which are not typically used for palliative purposes. The incidence of interstitial lung disease (ILD) related to TKI use is low. In a review of 21 clinical trials including 1468 patients treated with EGFR TKIs, the incidence of grade 3 and higher pulmonary toxicity was about 2% [82]. While most studies have not reported significant increases in pulmonary or esophageal toxicity with concomitant TKI and radiation use [82,83,84,85,86,87], comprehensive data on optimal timing and potential unexpected toxicities remain limited, and some reports warn of unexpected toxicity of such combinations, especially for the lung [88,89,90]. Therefore, to mitigate the risk of severe toxicity, TKIs are often temporarily discontinued during radiotherapy. Given the short half-life of most TKIs (less than 48 h), these agents are typically halted 2–4 days before starting radiotherapy, with treatment ideally concluding as quickly as possible. TKI therapy may resume the day after radiotherapy if no grade 2 or higher toxicity emerges [91]. Consequently, short courses of ablative radiotherapy, such as SBRT or hypofractionated schedules, are preferred whenever feasible. In cases where radiotherapy is initiated upon the first progression under first-line EGFR TKI, ALK, or ROS1 inhibition, the molecular agent is generally continued to prevent the flare phenomenon. Consequently, interruptions in administration are minimized. For this reason, TKIs are sometimes not interrupted during radiotherapy based on studies demonstrating the feasibility of such an approach. Retrospective comparisons of patients who continued osimertinib during radiotherapy versus those who paused it did not reveal significant changes in pneumonitis risk with concomitant use of these modalities [92]. An ongoing randomized phase III trial (NORTHSTAR, NCT03410043) aims to determine whether adding local treatment (primarily radiotherapy) to the new generation of TKI (osimertinib) in stage III and IV (oligo- and polymetastatic) NSCLC provides a survival benefit and does not discontinue osimertinib during radiotherapy [93]. This trial is expected to confirm the safety of concurrent TKI and radiotherapy use. 

Anti-angiogenic agents like bevacizumab, a vascular endothelial growth factor (VEGF) antibody used in NSCLC treatment, possess radiosensitizing properties. By inhibiting VEGF, they normalize pathological vessels in tumors, decrease tumor hypoxia, and enhance radiosensitivity. Radiosensitivity is further enhanced by inhibiting DNA double-strand break repair in endothelial cells using anti-angiogenic agents [94,95]. However, the potent radiosensitizing properties of bevacizumab preclude its use in combination with radiotherapy, as several prospective trials were prematurely closed due to major toxicities associated with these combinations. Apart from increased risks of pneumonitis and esophagitis, there is also an elevated risk of fatal bleeding and the formation of bronchoesophageal fistulas [96,97,98,99]. This increased risk can be explained by vascular damage caused by bevacizumab. Reports indicate an increased risk of fatal bleeding when using radiation for central lesions in patients receiving bevacizumab [96,99]. In a group of 108 patients receiving SBRT for central lesions, two of four toxic deaths resulted from pulmonary hemorrhage in patients previously treated with bevacizumab [100]. Central tumor locations are typically targeted in palliative thoracic radiotherapy, so caution is paramount for such patients. The start of radiotherapy should be postponed in these cases, except in emergency situations. Even in emergencies, large fractional doses should be avoided.

## 7. Conclusions

The effectiveness of palliative thoracic radiotherapy for NSCLC and the basic principles of its use in clinical practice were established during an era when systemic treatment was limited to one to three lines of cytotoxic chemotherapy, and modern radiotherapy technologies such as 3D-CRT, IMRT, or SBRT were either unavailable or their cost and accessibility prevented their use in patients with a poor prognosis. The finding established at that time that there is no difference in symptom relief between single-dose and protracted radiation schedules for symptom relief remains undisputed. Significant reduction in or even remission of symptoms in some patients with poor PS may lead to an improvement in PS, making it possible for them to access modern personalized systemic treatments, which are typically reserved for patients with good PS. For such patients, as well as in emergency situations, the radiotherapy technique, dose, and fractionation are of lesser importance; the primary goal is rapid symptom relief.

The increase in survival rates, coupled with the growing availability of treatment options for patients with good PS not suitable for radical radiotherapy, necessitates applying similar principles for palliative radiotherapy as those used in curative approaches to avoid late side effects and prevent relapse at the irradiated site. Consequently, higher doses and modern radiotherapy technologies should be considered. However, in thoracic palliative radiotherapy, the central tumor location and large treatment volumes often preclude the use of high ablative doses with SBRT, making moderate hypofractionation delivered with new technologies more suitable.

The combination of thoracic palliative radiotherapy with molecular targeted therapy or immunotherapy remains an area of ongoing research. All factors, including patient prognosis, access to potentially effective treatments, expected outcomes, and toxicity risks, should be thoroughly assessed and discussed with oncologists to define comprehensive palliative radiotherapy strategies. Decisions regarding radiation timing, treatment volumes, the continuation versus discontinuation of immunotherapy or targeted therapy, and alternative treatment modalities should ideally be made within a multidisciplinary tumor board setting.

## Figures and Tables

**Table 1:** The use of palliative schemes of thoracic radiotherapy.

Symptomatic Patients	Asymptomatic Patients
For alleviation of symptoms caused by intrathoracic growth of the primary or nodal metastases: Cough Dyspnea Hemoptysis Pain Dysphagia Pain Superior venae cavae syndrome Others (hoarseness, compression of nerves)	As prevention of the occurrence of symptoms
	For frail patients not amenable for curative doses of radiotherapy or systemic treatment
	For patients who cannot receive curative doses of radiotherapy due to the extent of the disease in the chest
	For oligoprogression during immunotherapy or targeted therapy and the extent of the disease preventing the use of high radiotherapy doses

**Table 2:** The advantages and disadvantages of respective radiation techniques for palliative thoracic radiotherapy.

Technique of Radiotherapy	Advantages for Palliative Thoracic Radiotherapy	Disadvantages for Palliative Thoracic Radiotherapy
Two-dimensional radiotherapy	Quick planning and delivery of usually two opposite fields. Suitable for emergency treatment or for patients in poor PS badly tolerating long procedures. Suitable for treatment with low total dose. Cheap and suitable in limited resources setting.	Currently, two-dimensional planning is commonly replaced with computerized 3D planning in radiotherapy departments; such techniques may not be longer in use; thus, a notion of cheaper and faster treatment may not be longer valid. Unsuitable for schedules with higher total doses with BED of about 30 Gy and higher because of increased esophageal toxicity
3D-CRT	Current equipment of radiotherapy departments makes 3D-CRT the most commonly used technique in this indication. Currently, it is easy and fast to use this technique for palliative purposes as well. Allows for accurate dose calculation for critical structures and, in selected cases, for a reduction in the dose given to organs at risk.	In selected cases, when high doses of radiotherapy are given or it is a special need for protection of critical structures, the use of more complex techniques, like IMRT or SBRT, may be preferred.
Endobronchal brachytherapy	Suitable for endobronchial lesions. Rapid relief of symptoms caused by tumors located in proximal bronchi, like dyspnea, recurring pneumonia, cough, or hemoptysis. Rapid dose fall-off outside the airways; suitable for patients with impaired lung function. Recommended for limited endobronchial recurrence after EBRT.	No benefit compared with EBRT as an up-front treatment. Not suitable for large lesions or nodal disease. Contraindicated for tumors located in close proximity to pulmonary arteries, especially if cavitated. Requires some expertise from the physician. Invasive procedure, risk of non-compliance from the patient.
IMRT (including V-MAT)	Used in cases with relatively good prognoses, especially in asymptomatic patients. When a high dose of radiotherapy is given for reducing side-effects or in selected cases with a need for special protection of organs at risk. V-MAT reduces treatment time and may be considered for some patients with limited compliance or tolerance of long treatment times.	Expensive Long in preparation and delivery Unsuitable for emergency cases No proven benefit over standard techniques for lower doses (like 20 Gy in 5 fractions).
SBRT	When ablative doses are decided (favorable cases) providing, it is technically feasible. Rapid dose decrease outside target reduces treatment toxicity. Short total treatment time allows for short discontinuation (if decided) of immunotherapy or targeted therapy or start of appropriate systemic treatment without unnecessary delay.	Unsuitable for large and centrally located lesions, which are usually subject to palliative thoracic radiotherapy. Long treatment preparation and delivery: unsuitable for emergency and suffering patients.

3D-CRT—three-dimensional conformal radiotherapy; PS—performance status; BED—biological effective dose; EBRT—external beam radiotherapy; IMRT—intensity-modulated radiotherapy; V-MAT—volumetric modulated arc therapy; Gy—gray; SBRT—stereotactic body radiotherapy.
